# The magnitude and associated factors of immune hemolytic anemia among human immuno deficiency virus infected adults attending University of Gondar comprehensive specialized hospital north west Ethiopia 2021 GC, cross sectional study design

**DOI:** 10.1371/journal.pone.0274464

**Published:** 2022-10-06

**Authors:** Samuel Sahile Kebede, Aregaw Yalew, Tesfaye Yesuf, Mullugeta Melku, Getachew Mesfin Bambo, Berhanu Woldu

**Affiliations:** 1 Department of Medical Laboratory Sciences, College of Medicine and Health Sciences, Mizan Tepi University, Tepi, Ethiopia; 2 Department of Hematology and Immunohematology, School of Biomedical and Laboratory Sciences, University of Gondar, Gondar Ethiopia; 3 Department of Internal Medicine School of Medicine, University of Gondar, Gondar, Ethiopia; 4 College of Medicine and Public Health, Flinders University, Adelaide, Australia; Universitas Indonesia Fakultas Kedokteran, INDONESIA

## Abstract

**Background:**

Immune hemolytic anemia commonly affects human immune deficiency infected individuals. Among anemic HIV patients in Africa, the burden of IHA due to autoantibody was ranged from 2.34 to 3.06 due to drug was 43.4%. IHA due to autoimmune is potentially a fatal complication of HIV which accompanies the greatest percent from acquired hemolytic anemia.

**Objective:**

The main aim of this study was to determine the magnitude and associated factors of immune hemolytic anemia among human immuno deficiency virus infected adults at university of Gondar comprehensive specialized hospital north west Ethiopia from March to April 2021.

**Methods:**

An institution-based cross-sectional study was conducted on 358 human immunodeficiency virus-infected adults selected by systematic random sampling at the University of Gondar comprehensive specialized hospital from March to April 2021. Data for socio-demography, dietary and clinical data were collected by structured pretested questionnaire. Five ml of venous blood was drawn from each participant and analyzed by Unicel DHX 800 hematology analyzer, blood film examination and antihuman globulin test were performed to diagnosis of immune hemolytic anemia. Data was entered into Epidata version 4.6 and analyzed by STATA version 14. Descriptive statistics were computed and firth penalized logistic regression was used to identify predictors. P value less than 0.005 interpreted as significant.

**Result:**

The overall prevalence of immune hemolytic anemia was 2.8% (10 of 358 participants). Of these 5 were males and 7 were in the 31 to 50 year age group. Among individuals with immune hemolytic anemia, 40% mild and 60% moderate anemia. The factors that showed association were family history of anemia (AOR 8.30 at 95% CI 1.56, 44.12), not eating meat (AOR 7.39 at 95% CI 1.25, 45.0), and high viral load 6.94 at 95% CI (1.13, 42.6).

**Conclusion and recommendation:**

Immune hemolytic anemia is less frequent condition in human immunodeficiency virus infected adults, and moderate anemia was common in this population. The prevalence was increased with a high viral load, a family history of anemia, and not eating meat. In these patients, early detection and treatment of immune hemolytic anemia is necessary.

## Introduction

### Background

In HIV infection, hematological parameters are mostly affected because of the viral effect on all lineages of blood cells and the immune system. The most typically affected hematological profiles are leukocytes, erythrocytes, and platelets [1]. Anemia is the most common hematological problem, affecting about 30% of asymptomatic and 75–80% of symptomatic HIV infections. From severely anemic individuals around 53% of HIV-infected adults may be caught by death [2, 3]. The most frequent anemia in HIV patients is normocytic normochromic type [4].

Immune hemolytic anemia (IHA) is normocytic or macrocytic normochromic anemia that occurs due to antibodies formed against one or more antigenic constituents of the individual’s tissues and results in the destruction of the erythrocyte. The main causes of IHA are primary or idiopathic and secondary by different underlying causes [5]. It can be classified as autoimmune hemolytic anemia (AIHA), alloimmune hemolytic anemia, and drug-induced hemolytic anemia (DIHA) [6]. Alloimmune hemolytic anemia occurs when antibodies are produced against red cells from another individuals, in transfusion, abortion and pregnancy [7]. AIHA results from the autoantibodies secondary to malignancies, autoimmune disorders and genetically [6]. It can be also classified as warm IHA, cold IHA and mixed IHA according to temperature of the reaction [5, 8].

In HIV patients IHA occurs due to Viral binding by HIV immune complexes to erythrocytes by one of three mechanisms. The mechanisms include the binding of complement-opsonized immune complexes via complement component receptor 1 (CR1), direct virus binding in a complement-dependent manner, but without the need for specific antibodies, and a third mechanism in which complement was not required at all. In absence of specific antibodies virus directly transported by erythrocyte surface during primary infection through duffy antigen [9, 10].

In the development of IHA by antigen-antibody complexes, the viral accessory protein negative factor plays a critical role in the pathogenesis of HIV-associated hematopoietic dysfunction. These factors affect the clonogenic potential of hematopoietic stem cells down modulates host cell receptors like a cluster of differentiation and major histocompatibility complex (MHC-1) molecules. It facilitates the transformation of infection into disease, increase viral infectivity, and increasing immunogenicity of the viral antigen mimic cell [1, 11, 12].

In addition to direct viral effect, it also occurs due to indirect effects of chronic generalized immune activation by HIV infection. These induce production of autoantibodies owing to the structural antigen similarity between the viral proteins and self-antigens [13]. The progressive decline of helper T-cell (CD4) caused by direct killing of infected cells that results from molecular mimicry of alloantigen with self, antiretroviral therapy (ART) drugs and opportunistic pathogens [14].

The autoimmune manifestations of HIV infection that cause IHA can be increased cytotoxic cell activity, increased expression of autoantigens, and alteration of erythrocyte surface antigen by a virus, and a cross-reaction between antibody induced by an infectious agent against erythrocyte surface antigen [15–18]. The factors that contribute to IHA includes increased viral load, tuberculosis, poor nutrition [19], family history of hemolytic anemia, neoplasia, unmatched blood transfusion, infection, and ART drugs [5, 20]. There is a variation of burden depending on the stage of HIV disease, sex, age, pregnancy status, history of abortion, and adherence to ART [21].

Immune hemolytic anemia in HIV-positive patients is a serious complication that occurs mostly in advanced age and stage of acquired AIDS especially in female patients. In Africa among anemic HIV patients, the burden of IHA due to autoantibody was ranged from 2.34 to 3.06% [17, 22], and due to DIHA was 43.4% [23]. It causes fever, jaundice, dark-colored urine, weakness, dizziness, confusion, hepatosplenomegaly, tachycardia and heart murmur [24]. Its consideration is important if patients experience severe to moderate anemia with low CD4 count [17].

Immune hemolytic anemia causes highly severe form of anemia. The most commonly reported age groups were middle-aged adult patients. Patients with IHA had lower mean CD4, Hb, RBC count, positive direct antiglobulin test (DAT), higher immature reticulocyte fraction, and mean reticulocyte percent than the non-anemic patients [25]. But DAT negative doesn’t mean there is no IHA because it may not be positive in the case of leukemia patients, immune-suppressed individuals, and low proteins [26]. In other way, a positive DAT is not in all cases resulted from IHA, because overt hemolytic anemia and aplastic anemia with hemolysis might be positive [27].

The diagnosis of IHA depends on the presence of laboratory findings supporting hemolysis such as increase of serum lactate dehydrogenase, haptoglobin, and unconjugated bilirubin in addition to DAT. The peripheral blood smear changes that are used as an indicator for hemolysis include reticulocytosis, shistocytosis, bite cells, and spherocytosis [8, 17].

The attention to IHA in PLHIV is less than expected globally, particularly in Ethiopia, due to limited studies considering IHA among PLWHIVs. Some studies that were done concerned with IHA due to autoimmunity even if they did not use immature reticulocyte fraction for differentiation of other cause of hemolysis from immune-mediated ones. The IHA burden is not well studied as its effect on people’s health, especially HIV patients and there was a knowledge gap between ART clinicians and other health professionals [17]. Therefore the aim of this study was to determine the magnitude and associated factors of anemia in HIV infected adults attending UOGCSH, North West Ethiopia.

## Method and materials

### Study design and period

An institutional based cross-sectional study was conducted to determine the magnitude of IHA and associated factors among HIV infected adults attending UOCSH North West Ethiopia from March to April 2021.

### Population

#### Source population

All HIV-infected adult individuals attending ART clinic in UOCSH, North West Ethiopia.

#### Study population

All HIV-infected adult individuals attending ART clinic at UOCSH, during a time of data collection can be used as the study population.

### Inclusion criteria and exclusion criteria

#### Inclusion criteria

All HIV infected individuals who were greater than or equal to 15 years or older, who had a confirmed HIV infection upon follow-up at the UOGCSH, and who had a clinical data and laboratory data’s such as viral load and CD4 counts in record within last six month of data collection time were included in study.

#### Exclusion criteria

Individuals who had been seriously ill and unable to respond and give blood specimens were excluded from the study.

### Sample size calculation and sampling technique

The sample size for this study was calculated using the single population proportion formula, since no study done was on IHA we used 50% proportion with 95% confidence interval and 5% marginal error, and finally using population reduction formula since the total population was less than 10,000 then the sample size obtained was 358.

## Sampling technique

**Fig 1:**
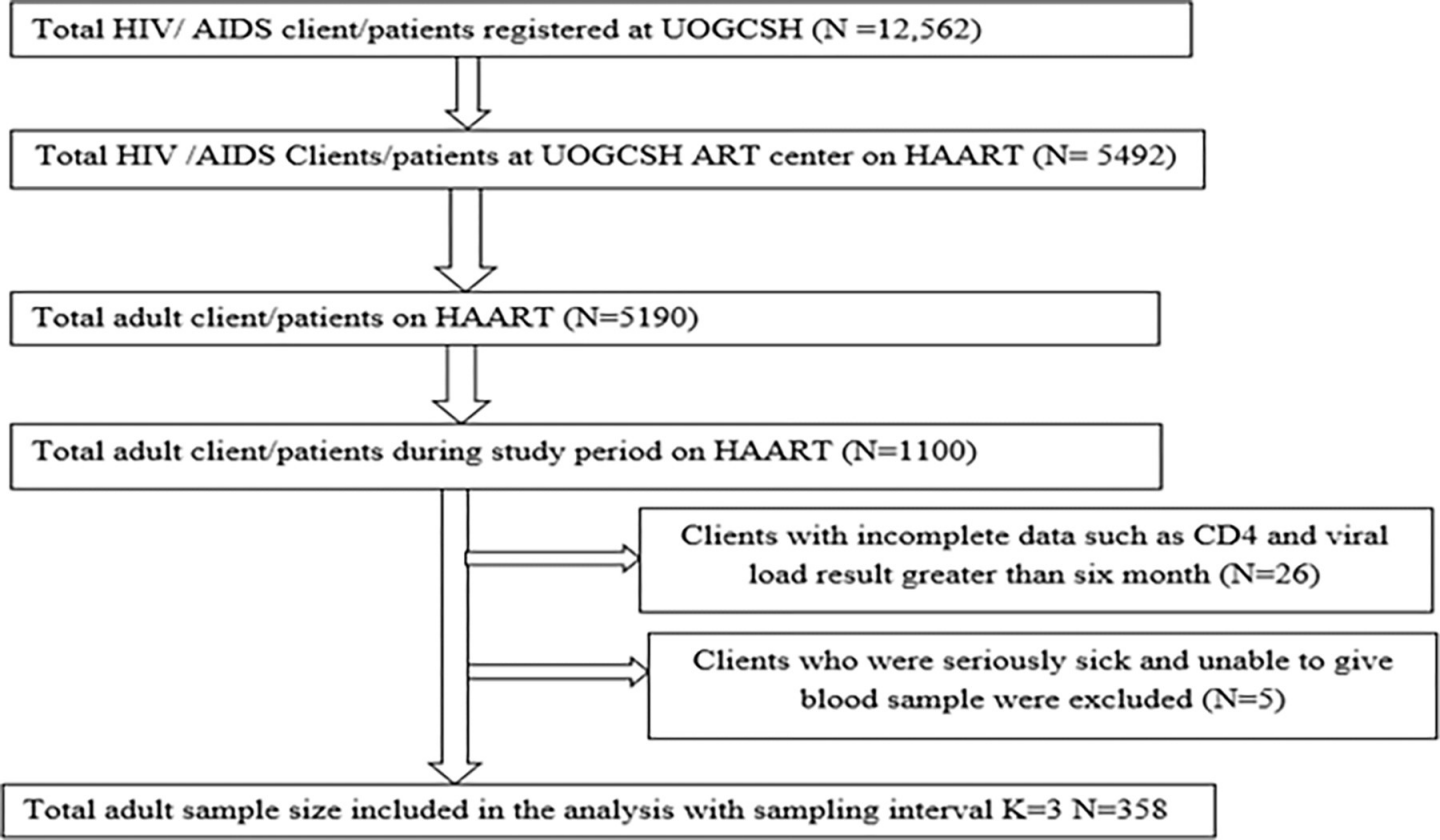
Schematic representation of the sampling procedure of HIV positive adults on HAART at University of Gondar comprehensive specialized hospital from March to April 2021.

A systematic random sampling technique was used to select study participants. The average number of HIV patients attending ART follow up every day and who gave a blood sample for viral load and CD4^+^ T-cell count concurrently were twenty five. During the two-month data collection period, 1100 PLWHIV were expected to visit the hospital for viral load and CD4^+^ T-cell count follow-up. The sampling interval (*K*) value was calculated by dividing the total number of HIV/AIDS patients during our study period by the sample size (1100/358 = 3). Then lottery method was used to select the first participant of three then taken by interval of three. The study subjects selected by every three individuals who are attending ART clinic of UOGCSH (Fig 1).

## Operational definition of variables

**Anemia** is condition at which adjusted Hb amount is less than 12 g/dl for female and less than 13 g/dl for male [2].

**DAT positive**: when agglutination is observed either after immediate centrifugation or after centrifugation that followed room temperature incubation of red cell suspension with antihuman globin reagent (83).

**Reticulocytosis:—**reticulocyte count greater than 2.5% or 158 x 10^9^ /L [28].

**Reticulocytopenia**:—reticulocyte cunt less than 0.5% or 18x10^9^ / L [28].

**IHA**:- is defined by a normocytic normochromic anemia or macrocytic anemia, which has hemolysis evidence in blood film such as spherocytosis or schistocytes, nucleated RBC, a reticulocyte count greater than 2.5% or immature reticulocyte fraction greater than 0.53 with positive DAT [29–32].

### Data collection tools and methods

#### Sociodemographic and clinical data collection

The data was collected by semi-structured questionnaire and collection was performed by trained expert nurses. The questionnaire had three parts including sociodemographic, clinical, and nutritional data which were related to IHA. The questionnaire was translated into the Amharic language. For sociodemographic data such as age, sex, residence, marital status, education, and religion were collected via face-to-face interview with study subjects. The clinical data’s such as history of abortion, CD4 result, viral load, neoplastic disease, opportunistic infection, history autoimmune disease and medication was extracted manually from participants’ records. For pregnancy, the female participants who were in age between 15–49 years old were screened by laboratory test of pregnancy and family history anemia was also requested by face to face interview (Annexes I, II in S1 File).

### Sample collection procedures and hematological analysis

#### Blood collection procedures

About 5ml of blood was collected with sterile syringe and needle by expert medical laboratory technologist into study participant code number labeled EDTA anticoagulant test tube. The collected blood sample was delivered to hematology laboratory for analysis of hematological parameters, DAT, and blood film preparation. The blood was transported to the hematology laboratory within 1 to 2 hours and the analysis to be performed. From the collected blood sample, hematological analysis was performed, then blood film were prepared from the remnant sample. Finally, DAT was performed on the rest blood sample (Annex V in S1 File).

#### Hematological analysis

The hematological analysis was performed on a blood sample in an EDTA anti-coagulated test tube to confirm the presence of IHA by following standard operating procedures. Hb measurement, reticulocyte count, immature reticulocyte fraction, RBC count, and RBC indices such as MCV, MCH, and MCHC were performed by using an automated hematology analyzer (Unicel DxH800, Danaher Corporation, Beckman Coulter, United States of America (USA)). Unicel DxH800 provides RBC count, reticulocyte count, immature reticulocyte fraction, and nucleated RBC on whole blood by impedance principle and leukocyte 5-part differential (Diff) and platelet by flow cytometery or light scattering principle [30, 32]. The blood sample is suspended in diluent and passes through the apparatus causing direct current resistance. Change in blood cell size is detected as the electrical pulse and blood cell count is measured by counting pulse (Annex V in S1 File).

#### Blood film examination

After hematological parameter performed, the remaining EDTA blood was used for blood film examination. A thin blood smear was prepared by wedge method by putting a drop of blood on the slide about 1-2cm from the end of the slide and making smear by another smooth-edged slide as spreader at an approximate angle of 30° on three fourth (¾) of the length of the slide. The prepared smear was air dried by placing the smear film side up on a staining rack. The dried smear was covered with filtered undiluted wright stain, left for 1minute, washed then dried, and examined by using oil immersion (100x objective) on the microscope. The morphology was examined by a trained technologist for the presence of features of hemolysis. In blood film the presence of schistocytes, spherocyte, bite cells, and nucleated RBC were evidence of hemolysis and in the meantime for cross-checking for morphology with analyzer result was performed (Annex V in S1 File).

#### Coombs test (DAT)

Three percent of washed blood suspension was used for direct ant globin test (DAT) for detection of coated antibody on the surface of red cells that results in immune hemolysis. Coombs test (DAT) was performed based on the principle of a hem agglutination test. Two Drops of the anti-globulin reagent added to two drops of the three percent of red cell suspension into the test tube. Polyspecific anti-human globulin antiIgG-C3d acts as a link between the antibodies and complement coating of neighboring RBC and induces agglutination. The test tube would be immediately centrifuged after thorough mixing and finally reading for presence of agglutination was examined microscopically then reported as positive or negative for DAT (Annex V in S1 File).

#### Immune hemolytic anemia (IHA)

Finally, Immune hemolytic anemia was diagnosed from the result of hematological parameters, blood smear, and coombs test results. It was defined as low Hb, normocytic or macrocytic red cell, feature of hemolysis on blood film such as bur cells, shistocytosis, spherocytosis, reticulocytosis, high immature reticulocyte fraction, and positive for direct anti-human globulin test. The test was finally confirmed for the presence of IHA by a laboratory technologist.

### Quality management of laboratory tests and data

#### Quality assurance for sociodemographic data

Before data collection, training was given to the data collectors to ensure the reliability and validity of data to reduce technical and observation bias. The questionnaires were tested (pretest) on randomly selected patients from the study site for reliability and validity before it was used for actual data collection. To check language translation information quality the translated questionnaire was reviewed by three individuals and retranslated back to the English language from Amharic. The validity of the information was checked again.

#### Quality control for hematology analyzer

Quality control for working equipment and reagents was ensured using standard controls as well as standard operating procedures. For Unicel DHX800 hematology analyzer normal background reading was checked daily and the performance was checked by low, normal, and high controls. The result of each test was properly recorded (annex v in S1 File).

#### Quality control for coombs test

The quality control was done for DAT, by using Rh-positive blood sample coated with an anti-D for positive control and negative control by Rh-negative blood were used. Then the result of both control was properly recorded (annex v in S1 File).

#### Quality control for microscopy and wright stain reagent

The preventive maintenance was performed for the microscope to prevent the entrance of abnormal artifacts in the morphological examination. The microscope was cleaned daily for quality examination of blood smear morphology and reticulocyte count. A microscopic smear review was performed to check functionality of microscope, quality of slide and staining by using previously examined and confirmed slides. To make quality staining, the solution was filtered before staining the smear. The quality control for wright staining solution was performed by using a patient sample with a normal MCV, MCH, MCHC and total white blood cell count (annex v in S1 File).

#### Data management and analysis

Data entry was entered into Epi data version 4.6 (Epidata, Inc. Redwood City, CA, United States) and analysis was done by using STATA (Software for statistics and data science) statistical software version 14 developed by StataCorp for data. Every day the collected data was checked for completeness and accuracy by the principal investigator. During the entry of data, it was cross-checked to assure the right data was entered and cleaned for accuracy. Descriptive statistics such as frequency, charts, tables, and percentages were used to summarize the data. The firth penalized logistic regression model was fitted to determine the associations of independent variables with outcome variables. For measure of association for variable was analyzed by bivariable firth penalized logistic regression model and those variables which had P value 0.2 were included in multivariable firth penalized logistic regression model to control the confounding factors. Then multivariable firth panelized logistic regression was computed for selected variables and the significance of association was determined and interpreted. Both Crude odds ratio (COR) and adjusted odds ratio (AOR) with their corresponding 95% confidence interval (CI) were used to see the strength of association between dependent and independent. A p-value <0.05 in multivariable firth penalized logistic regression model was considered statistically significant. The results were presented in words and tables. Based on the study result, conclusions and recommendations were done.

#### Dissemination of results

The study result would be submitted to Department of Clinical Hematology and immunohematology the School of BMLS and CMHS, UOG, and also the results would be submitted to the study site. The abstract would be submitted to local concerning bodies Such as EMLA and libraries. The result would be communicated with the research community through a presentation on conferences and publication on peer-reviewed reputable journals to communicate with the international community.

## Result

### Sociodemographic characters

The total number of participants in this study was 358. Of the total participants, 216 (62.1%) of them were females and the median age was 38 (interquartile range 33 to 45) years. Among the study participants 313 (87.43%) were Orthodox Christian, and 285 (79.61%) were urban residents. Of all participants 187(52.87%) were married followed by divorced, 90(25.14%) were government employee and 115(32.12%) had secondary school education level (Table 1).

**Table 1:** Sociodemographic characters of study participants for the magnitude of IHA and associated factors among HIV infected adults at UOGCSH March to April 2021 (N = 358).

Variables	Category	Frequency (N)	Percent (%)
Age	15–30	71	19.83
	31–50	245	68.44
	51–65	42	11.73
Gender	Male	142	39.64
	Female	216	60.33
Residency	Urban	285	79.61
	Rural	73	20.39
Religion	Orthodox	313	87.43
	Muslim	36	10.06
	Others ^**a**^	9	2.51
Marital status	Single	44	12.29
	Married	187	52.23
	Divorced	94	26.26
	Widowed	33	9.22
Occupation	Farmer	30	8.38
	House wife	88	24.58
	Merchant	90	25.14
	Government employee	90	25.14
	Others ^**b**^	60	16.76
Educational Status	No formal education	93	25.98
	Primary School	90	25.14
	Secondary school	115	32.12
	University/college	60	16.76

^a^ (other religion) = protestant, Jehovah’s Witness

^**b**^ (other occupation) = sex worker, laborer, carpenter, Non-government organization worker

### Clinical characteristics of the study participants

From the study participants, 117 (32.68%) of them had a history of comorbidity of HIV and opportunistic infections including bacterium tuberculosis, 33 (9.22%), had a family history of anemia and 66 (18.44%) had history of an autoimmune disease. Of all study participants, 24 (6.70%) of study participants had a history of neoplastic disease, and 278(77.65%) were on stage one of disease (AIDS). Among female study participants 2(0.93%) had history of pregnancy but none of them had abortion history in last four months (Table 2). Among study participants, 52.79% (189) drink coffee at least once a day, 94.13% (337) of them use (consume) meat in diet and 280 (78.21%) use green vegetables daily (Table 2).

**Table 2:** Clinical characters of study participants for the magnitude of IHA and associated factors among HIV infected adults at UOGCSH from March to April 2021.

Variables	Category	Frequency (N)	Percentage (%)
Pregnancy (n = 216)	Yes	7	3.27
	No	209	96.73
History of birth in last four month (n = 216)	Yes	2	0.93
	No	214	99.07
History of transfusion (n = 358)	Yes	34	9.50
	No	324	90.50
History of opportunistic infection including TB (n = 358)	Yes	117	32.68
	No	241	67.32
Family history of anemia (n = 358)	Yes	33	9.22
	No	250	69.83
	Unknown	75	20.95
Stage of AIDS (disease) (n = 358)	Stage 1	278	77.65
	Stage 2	60	16.76
	Stage 3 and 4	20	5.59
History of auto immune disease	Yes	66	18.44
	No	292	18.44
History of neoplastic disease (n = 358)	Yes	24	6.70
	No	334	93.30
HAART- regimen	AZT-containing	81	22.63
	Non AZT-	277	77.37
History of other medication	Yes	153	42.2
	No	205	57.8
Other medication (n = 205)	ant-TB	107	69.93
	antibiotics	20	13.07
	other medication	26	16.99
HAART duration	On 3 months and bellow	89	24.86
	On 6-months	245	68.44
	more than 6 month	24	6.7
Drink coffee per day	Less often per day	169	47.21
	At least once a day	189	52.79
	Less often per day	222	62.36
	At least once a day	134	37.64
Weekly use (consume) meat (N = 358)^a^	Less often per week	261	77.44
	At least once a week	76	21.23
	never	21	5.86
	At least once a day	78	21.79
Daily use green leafy vegetables	Less often per day	280	78.21

### Hematological and immunological profiles

Among the study participants, 101 (28.21%), 53 (14. 8%), 37 (10.34% and 10 (2.8%) had anemia, thrombocytopenia, leukopenia and pancytopenia, respectively. The study participants mean Hb level was 13.17 (95% CI 12.99, 13.36) g /dl, mean immature reticulocyte count was 0.37 (95% CI 0.36, 0.38) and mean relative reticulocyte count was 0.96% (95% CI 0.90% to 1.01%). Based on RBC morphology and red cell indices of anemic individuals, 62 (61.38%), 21 (20.8%), 18 (17.82%) had normocytic normochromic, microcytic hypochromic, and macrocytic anemia respectively. Of those anemic study participants 1(0.99%), 21 (20.80%), 79 (78.21%) had severe, moderate, and mild anemia respectively. Among anemic study participants 18(17.82%) had spherocyte and 24 (23.76%) had schistocytes and bite cells on peripheral morphology. From the total parameters IHA anemia can be defined based on decreased hemoglobin level with normocytic or macrocytic RBC, evidence of hemolysis increased reticulocyte count or immature reticulocyte fraction and positive for DAT. The individuals who met this criteria were 10(2.8%) (Table 3).

**Table 3:** Hematological and immunological profiles of study participants for magnitude of IHA in HIV infected adults at UOGCSH during March to April 2021 (N = 358).

Hematological and immunological profiles		Category	Frequency(N)	Percent (%)
RBC	Male (n = 142)	< 4.26 X10^12/^/L	45	12.6
		4.26–6.68 X 10^12^/L	94	26.25
		> 6.68X 10^12^/L	3	0.84
	Female (n = 216)	< 4.02X 10^12^/L	36	10.05
		4.02–6.15X 0^12^/L	178	49.72
		>6.15X 0^12^/L	2	0.56
Hemoglobin	Male (n = 142)	<13 g/dl	41	11.45
		13–18.76 g/dl	96	26.81
		>18.76 g/dl	5	1.4
	Female (n = 216)	<12 g/dl	60	16.8
		12–16.7 g/dl	151	42.18
		>16.7 g/dl	5	1.4
MCV		<85(fl)	24	6.7
		85–100 (fl)	273	76.26
		>100(fl)	61	17.04
MCH	Male (n = 142)	<26.6(pg.)	14	3.63
		26.6–33.3(pg.)	102	28.5
		>33.3(pg.)	26	7.26
	Female (n = 216)	25.8(pg.)	8	2.24
		25.8–32.8 (pg.)	156	43.6
		>32.8(pg.)	52	14.50
Leukocyte		<3.24 x 10^9^/L	37	10.34
		3.24–10.5 x 10^9^/L	309	86.31
		>10.5 x10^9^/L	12	3.35
Platelets	Male (n = 142)	<164X 10^9^/L	17	4.75
		164 -403X 10^9^/L	116	32.24
		> 6.15X 0^12^/L	9	2.51
	Female (n = 216)	<202.5 X 10^9^/L	36	10.05
		202.5–444.5 X 10^9^/L	174	48.6
		>274 X 10^9^/L	6	1.4
Reticulocyte count (n = 101)		<0.5%	12	11.88
		0.5–2.5%	75	74.25
		>2.5%	14	13.86
Immature reticulocyte fraction (IRF) (n = 101)		< 0.3	16	15.84
		0.3–0.53	74	73.30
		> 0. 53	11	10.89
Direct Coombs test (DAT) (n = 101)		Positive	19	18.81
		Negative	82	81.19
Viral load		≤ 1000	336	93.85
		>1000	22	6.15
Cluster of differentiation 4 (CD4)		<200	75	20.95
		≥200	283	79.05

**NB; Reticulocyte**, immature reticulocyte fraction, DAT were tested for anemic individuals

### Prevalence of immune hemolytic anemia

The overall prevalence of IHA (who met criteria for IHA) in this study was 2.80%. (95% CI 1.07, 4.50). The prevalence of IHA among HIV in patients the 15–30 years age group was 4.22% (n = 3), However, there was no IHA found in the age group greater than 50. Among individuals who had IHA, 6 (60%) of them had moderate, and the rest of them had mild anemia. However, there was no severe anemia case in a patient who had IHA in this study.

### Factors associated with immune hemolytic anemia

To determine the association between the IHA and independent variable bi-variable and multi-variable firth penalized logistic regression model was used. Based on the analysis variables with p value less than 0.2 in bivariable firth penalized logistic regression model included in multivariable analysis. Accordingly, vegetarianism (do not using meat in their diet), high viral load and family history of anemia showed significant association with IHA (Table 4).

**Table 4:** Bivariable and multivariable firth penalized logistic regression model analysis of factors associated with IHA among HIV infected adults from March to April 2021 (N = 358).

Variables	Category	IHA		COR at CI (95%)	AOR at CI (95%)	p-value
		Yes	No			
Age	15–30	3 (4.22)	68 (95.78)	1		
	31–50	7 (2.86)	238 (97.14)	0.61 (0.16, 2.25)		
	51–65	0 (0.00)	42 (100)			
Residence	Urban	5 (1.75)	280 (98.25)	1	1	
	Rural	5 (6.85)	68 (93.15)	4.11 (1.27,14.4)	2.58 (0.59,11.20)	0.84
Gender	Male	5 (3.52)	137 (96.48)	1		
	Female	5 (2.32)	211 (97.68)	0.65 (0.19, 2.16)		
Education	No formal education	3 (3.30)	90 (96.70)	0.95 (0.23,3.98)		
	Primary School	3 (3.22)	87 (96.78)	0.99 (0.23,4.11)		
	Secondary school	4 (3.48)	111 (96.52)	1		
	University/college	0 (0.00)	60 (100.00)			
History of opportunistic infection	Yes	4 (3.54)	113 **(**96.46**)**	1.43 (0.42, 4.87)		
	No	6 (2.49)	235 **(**97.51**)**	1		
Medication	Ant-TB	4 (3.74)	103 **(**96.26**)**	1.56 (0.44, 5.0)		
	Other medication	1(2.17)	45(97.87)	1.22 (0.19, 7.69)		
	Never	5(2.38)	205(97.62)	1		
History of auto immune disease	Yes	3(4.54)	63(95.45)	0.47(0.13,1.74)		
	No	7(2.4)	285(97.60)	1		
Meat consumption	At least per weak	2 (2.6)	73 (97.4)	1		
	Less often / weak	5 (1.90)	257(98.1)	0.62(.13,2.86)	0.98(0.19, 5.0)	
	Never	3(14.28)	18(85.71)	5.56(1.1, 30.23)	8.18(1.60, 41.74)	0.01
Daily use green leafy vegetables	Less often per day	8 (2.86)	272 **(**97.14**)**	1.04 (.25, 4.4)		
	At least per day	2 (2.56)	76 **(**97.44**)**	1		
Coffee drinking	Less often per day	8 (2.98)	161 **(**97.02**)**	3.95 (0.95,4.87)	2.29(0.51, 10.19)	0.38
	At least per day	2 (1.06)	187 **(**98.94**)**	1		
Tea drinking	Less often per day	4 (1.80)	218 **(**98.20**)**	0.40 (0.12,1.39)	0.51 (0.12, 2.17)	0.22
	At least per day	6 (4.48)	128 **(**95.52**)**	1		
CD4	<200	4 (5.30)	71 **(**94.70**)**	2.62(0.77,8.93)	0.89 (0.15, 5.01)	0.93
	≥ 200	6 (2.12)	277 **(**97.88**)**	1		
Stage of disease	Stage 1	6 (2.16)	272 **(**97.84**)**	1		
	Stage 2	3 (5.005	57 **(**95.00**)**	2.53(0.74,8.58)	1.48 (0.37, 5.93)	0.2
	Stage III and IV	1 (5.00)	19 **(**95.00**)**	3.22(0.51, 20.18)	1.73 (0.08, 39.31)	0.16
Viral load	>1000	5 (6.60)	71 **(**93.40**)**	19 (5.25, 67.4)	6.94(1.13, 42.6)	0.04
	≤ 1000	5 (1.77)	277 **(**98.23**)**	1	1	
HAART regimen	AZT-containing	4 (4.94)	77 **(**95.06**)**	2.40(0.70,8.17)	1.44 (0.37, 5.61)	0.61
	Non-AZT	6 (2.17)	271 (97.83**)**	1		
Family history of anemia	Yes	3(9.09)	30(91.01)	5.12(1.29, 20)	8.3(1.56,44.12)	0.01
	No	5(2)	245(98)	1		
	I do not know	2(2.6)	73(97.4)	1.51(.33, 6.92)	1.790(.375, 8.55)	0.45

At P value <0.05

AZT: Ziduvidine, CI: Confidence Interval, HAART: Highly active antiretroviral therapy AOR: Adjusted odd ratio, COR: Crude odd ratio, IHA: Immune Hemolytic Anemia

## Discussion

Human immuno deficiency virus infection triggers anemia, which is most likely caused by HIV infection of stromal cells and hematopoietic stem cells. In HIV infection, the commonly affected hematological parameters are leukocyte, erythrocyte, and platelets due to viral effect on all lineages of blood cells and immune system [1]. Of all hematological abnormalities anemia is common among HIV patients. The pathophysiology of anemia includes decreased production, increased destruction, and increased loss due to hemorrhage [3]. IHA is a type of anemia caused by immune mediated destruction of RBC by antibodies against erythrocyte antigens. It is characterized by normocytic normochromic or macrocytic anemia with hemolysis evidence in -blood film, reticulocytosis or high immature reticulocyte fraction and positive for DAT [29–31].

The overall prevalence of IHA among HIV-positive adults was 2.80% (95% CI 1.08%, 4.50%), The prevalence was in agreement with studies conducted in Addis Ababa (2.34%) [17] and Benin (Nigeria) (3.06%) [22]. However, it is higher than that of study done in Lagos (Nigeria) (0%) (38). The difference might be resulted from the variation in the defining IHA. The study in Lagos (Nigeria) was the defined IHA by using a reticulocyte count, hemoglobin level and coombs test only, without using immature reticulocyte count. But in this study, immature reticulocyte fraction was used for diagnosis of IHA. Reticulocytopenia is common in HIV patients, this causes misdiagnosis of IHA in HIV patients. The immature reticulocyte fraction is the best parameter for diagnosis of IHA in HIV patients, because it increases in case of IHA regardless of HIV status [33, 34].

According to this study, among individuals who had IHA, 60% of them had moderate and 40% of them had mild anemia. This finding did not agree with the study in Addis Abeba [17]. The study indicated 22.2% of them had severe and 33.3% of them had moderate anemia. This variation might be due to the advancement of ART medication from AZT-based regimen especially navirapine to new advanced regimens with reduced adverse effect such as dolutegravir based regimen. Even though the mechanism is not fully elucidated, IHA occurs as part of drug rash with eosinophilia and systemic symptoms syndrome in the presence of drugs (navirapine). The drug dependent antibody mediated hemolysis appears within two weeks after initiation of the drug, whereas the patient presents with rapidly progressing IHA. But dolutegravir did not cause anemia and dolutegravir-containing regimen demonstrated a high virologic efficacy. This might protects patients from developing severe anemia [35, 36].

The determinant factors which shown significant association with IHA were family history of anemia (AOR 8.30 at 95% CI 1.56, 44.12), vegetarian life style (not consuming meat) (AOR 7.39, CI, 95% 1.13, 42.6), and high viral load (AOR of 6.94 at 95CI % (1.13, 42.6).

In this study, individuals whose families had a history of anemia were 8.30 times more likely to develop IHA than their counterparts (AOR 8.30, 95% CI 1.56, 44.12). This might be due to the presence of study participants with family history of IHA. IHA might be caused by a fundamental defect in the immune system that inhibits the immune system from establishing a proper homeostatic mechanism. This disorder appears to be passed down in families which block erythrocyte immune homeostasis. The patients with hereditary spherocytosis, who had naturally occurring autoantibodies directed against different membrane proteins. This antibody makes the reaction with the surface antigen of erythrocyte and results in immune mediated hemolytic anemia [37, 38].

According to finding of this study, vegetarians or peoples who did not eat meat were 7.39 times (AOR 7.39, CI, 95% 1.13, 42.6) at risk of developing IHA than individuals who eat meat in their diet. This study agrees study done in Shalla, [Ethiopia] [39], Vietnam [40], and Pakistan [41], which reported that anemia was higher among individuals who did not use meat and animal products. Lack of meat in diet results in vitamin B12 deficiency which causes impaired immune system activity such as decreased lymphocyte especially CD8, natural killer cells, lymphokine activated killer cells and an increase in the CD4/CD8 ratio [42, 43]. The impaired levels of the immune activity is associated with higher risk of HIV disease progression and increased viral replication. The increased virus causes viral protein induced immune activation which might result in IHA in HIV patients [44].

In this study, individuals whose viral load greater than 1000 copies/μl were 6.94 times more likely to develop IHA than individuals whose viral load less than 1000 copies/ μl (AOR of 6.94 at 95CI % (1.13, 42.6)). The higher viral load is indicative of poor suppression of viral quantity. This might be occurred due to the positive correlation between plasma HIV ribonucleic acid levels and both CD4+ T-cell activation and CD8+ T-cell activation levels [45]. Virus induces IHA by HIV binding with erythrocytes, then causes immune activation, dysregulation of T and B cells, immune intolerance and expression of auto antigens similar to virus [9, 10]. The structural antigen similarity between HIV proteins and RBC antigens can induce autoantibody production. Moreover, the presence of viral negative factor protein induces autoimmune responses by cross-reaction of specific viral antigens with self-proteins through the stimulation of auto-reactive T cells [45]. The circulating autoantibodies to RBC and host red cell with increased immunogenicity finally these results antibody-mediated hemolysis or IHA [11, 45, 46].

## Strength and limitation

### Strength of the study

In this study hematological analysis, such as reticulocyte count, mean reticulocyte volume and immature reticulocyte fraction were performed by automation. This study also attempted to describe associated factors of IHA in addition to prevalence.

### Limitation

The first limitation of this study was a cross-sectional nature of its design, it did not allow us to observe causality in the relationship between IHA and its associated factors, as it is temporal association. The other limitation was, this study was not included DAT negative IHA which requires latest technology such as gel technology and molecular methods. The serum lactate dehydrogenase, haptoglobin, and unconjugated bilirubin were not tested for additional evidence of hemolysis.

## Conclusion and recommendation

### Conclusion

According to the findings of this cross-sectional study IHA in HIV patient is rare public health problem. This finding revealed that IHA was significantly associated with vegetarianism, family history of anemia and high plasma viral load.

### Recommendations

The ART clinicians were recommended to focus on viral load to monitor disease progress and give attention for IHA. IHA screening test has to be done specifically before blood cell transfusion in HIV patients. We recommend HIV patients to use meat in their diet to protect themselves from vitamin B12 deficiency induced IHA. Individuals who had family history of anemia should be screened for IHA. Additionally, we recommend that additional study to be done by using sensitive and specific advanced technology products like flow cytometery and advanced molecular testes which also help to quantify amount of RBC bounded antibodies to know the probability for hemolysis. It is better for researcher in hematology area, give attention to set the reference interval of immature reticulocyte fraction and mean reticulocyte volume. Even though it is less frequent IHA diagnosis needs prior identification to minimize severity and burden of disease because it may result in fatal condition. We suggest policy makers to develop guideline for HIV patient by considering IHA and work to make screening tests for IHA to be available in every ART clinic across the country.

## Declarations

### Ethical approval and consent to participate

#### Ethical considerations

The study was carried out after receiving ethical approval from the University of Gondar college of medicine and health sciences (CMHS), school of biomedical and laboratory science research, and ethical review committee (Reference number SBLS/2750). All activity in this research work was based on Helsinki declaration. Furthermore, support and permission letter were secured from UOGSCH. In addition, following an explanation of the purpose, the benefits and the possible risks of the study, written informed consent was taken from a parent/legal guardian and assent was sought from children before commencement of the study. It was made clear that participation in the study were purely on a voluntarily basis and refusal was possible. To ensure confidentiality of data, study participants were coded by using unique codes, and only authorized persons were accessing the collected data. The study participant’s with abnormal findings were linked to the physicians who are working at the ART clinic for proper patient care.

### Availability of data and materials

All relevant data are available within the manuscript. In case of need, the data that support the findings of this study are available from the corresponding author on reasonable request.

## Supporting information

S1 File (DOCX)

## Acronyms/abbreviation

AIDS: Acquired Immunodeficiency Syndrome AIHA: Autoimmune Hemolytic Anemia
ART: Anti-Retroviral Therapy
AOR: Adjusted Odd Ratio
AZT: Azidothymidine
COR: Crude Odds Ratio
CD: Cluster of Differentiation
CI: Confidence Interval
DAT: Direct Antihuman Globulin Test
DIHA: Drug Induced Hemolytic Anemia
EDTA: Ethylene diamine tetra acetic acid
HAART: Highly Active Anti-Retroviral Therapy
Hgb: Hemoglobin
HIV: Human Immune deficiency Virus
IHA: Immune Hemolytic Anemia
MCH: Mean Cell Hemoglobin
MCHC: Mean Cell Hemoglobin concentration
MCV: Mean Cell Volume
PLWHIV: Peoples Living with Human Immune deficiency Virus
RBC: Red Blood Cell, TB: Tuberculosis
UOGCSH: University of Gondar comprehensive specialized hospital
