# Supplementary material for: The magnitude and associated factors of immune hemolytic anemia among human immuno deficiency virus infected adults attending University of Gondar comprehensive specialized hospital north west Ethiopia 2021 GC, cross sectional study design
Source: PLoS One. 2022 Oct 6;17(10):e0274464. doi: 10.1371/journal.pone.0274464 (PMC9536541; doi:10.1371/journal.pone.0274464)
Supplement: S1 File — (DOCX) [file pone.0274464.s001.docx]

. Annexes

## **Annex I: English Version Information sheet and Consent form**

**Part I:** **Information sheet**

**Introduction**: My name is Samuel Sahile I am currently MSc student of university of Gondar department of clinical hematology and immune hematology I am going to conduct a survey on magnitude of IHA and associated factors in HIV positive patients.

**Study title**: Magnitude and associated factors of immune hemolytic anemia among human immuno deficiency virus infected adults at university of Gondar comprehensive specialized hospital North West Ethiopia from March – April 2021 GC.

**Objective of the study:** the aim of this study is to determine and associated factors of IHA among HIV infected adults at university of Gondar specialized referral hospital at 2021 GC.

**Benefit of this study:** Conducting this study will be used to advance the diagnosis of anemia in these individuals and know the current status of the problems. If you have anemia especially IHA you will be linked to ART clinicians with your laboratory results for additional diagnosis and treatment of disease. The study output (result) will be used for planning health programs and policy makers for reduction of anemia as well as IHA. You understand that you will not get any financial benefit. Your cooperation and willingness to the study will be very helpful in understanding current prevalence of problem.

**Risks of this study for participants**: There may be little pain during blood sample collection but do not cause any health harm or long live abnormality.

**Rights and privacy**: Your name will not be written in the form and I assure you that all the information will be kept strictly confidential. Your participation is voluntary based and you are not obligated to participate. You should know that the information and blood sample that going to be used for this study only. All the information given for the study and the results are confidential. If you are not comfortable please feel free to refuse. Therefore, with full understanding of the situations you agree to give the entire necessary information **a**nd blood sample for laboratory analysis.

**Person to contact;** please direct any questions or problems you may encounter during this study to the principal investigator. Address +251917768545/samissahile45@gmail.com

**Informed consent**

I participant undersigned the purpose of the study titled as magnitude of immune hemolytic anemia and associated factors among HIV positive adults at university of Gondar compressive specialized hospital. I have been informed there is no harm except little discomfort during sample collections. I have been informed that other people will not know my results. I understand that there is no benefit to me personally apart from clinical service I get from these results .I have been told that participation in this study is voluntary and I may refuse to be in the study. The study has been explained to me in the language I understand. I give consent to participate after a clear understanding of the objectives and conditions of the study.

Participant’s name ----------------------------Signature----------------------- Date: -----------------------

Data collector name: --------------------------- Signature: ---------------------- Date: -----------------

Name of investigator _____________Signature ___________Date of investigation __________

**Informed assent**

I guardian undersigned the purpose of the study titled as magnitude of immune hemolytic anemia and associated factors among HIV positive adults at university of Gondar compressive specialized hospital. I have been informed there is no harm except little discomfort during sample collections. I have been informed that other people will not know my patients results. I understand that there is no benefit to me personally apart from clinical service he/she get from these results .I have been told that participation in this study is voluntary and I may refuse as my patient want to refuse to be participant in the study. The study has been explained to me in the language I understand. I give consent to participate after a clear understanding of the objectives and conditions of the study.

Guardians’ name --------------------------------------------

Relationship to participant: -----------------Signature----------------------- Date: ------------------------

Data collector name: --------------------------- Signature: ---------------------- Date: -----------------

Name of investigator _____________Signature ___________Date of investigation __________

ID.No:______________

| Socio –demographic ,clinical and nutritional information of HIV Positive adult patients | | | |
| --- | --- | --- | --- |
| S no | Socio –demographic questions please encircle in correct information you have. | | |
| 01 | How old are you? Age in years |  | |
| 02 | Sex | Male | 1 |
|  |  | Female | 2 |
| 03 | Where do you live?(Residence) | Urban | 1 |
|  |  | Rural | 2 |
| 04 | What is your occupation? | Farmer | 1 |
|  |  | House wife | 2 |
|  |  | Merchant | 3 |
|  |  | Governmental employee | 4 |
|  |  | Labor worker | 5 |
|  |  | Others | 6 |
| 05 | Religion | Orthodox | 1 |
|  |  | Muslim | 2 |
|  |  | Protestant | 3 |
|  |  | Others | 4 |
| 06 | How much is your monthly income? | |  |
| 07 | What is your educational Status? | No formal education | 1 |
|  |  | Primary School | 2 |
|  |  | Secondary school | 3 |
|  |  | University/college | 4 |
| 08 | Marital status | Single | 1 |
|  |  | Married | 2 |
|  |  | Divorced | 3 |
|  |  | Widowed | 4 |

B) Clinical data of participant

| 09 | If Q2 is female | Pregnant | 1 |  |  |  |
| --- | --- | --- | --- | --- | --- | --- |
|  |  | Not pregnant | 2 |  |  |  |
| 10 | If question 2 is female have you given birth within 4 month | Yes | 1 |  |  |  |
|  |  | No | 2 |  |  |  |
| 11 | History of transfusion | Yes | 1 |  |  |  |
|  |  | No | 2 |  |  |  |
| 12 | If yes in No 9, how long it was?(in month) |  |  |  |  |  |
| 13 | Have you past history TB? | Yes | 1 |  |  |  |
|  |  | No | 2 |  |  |  |
| 14 | Have you taken any medication out of ART? | Yes | 1 |  |  |  |
|  |  | No | 2 |  |  |  |
| 15 | If question number 14 yes which type |  | |  |  |  |
| 16 | Family history of hemolysis(anemia) | Yes | 1 |  |  |  |
|  |  | No | 2 |  |  |  |
|  |  | I don’t know | 3 |  |  |  |
| 17 | History of auto immune disease | Yes | 1 |  |  |  |
|  |  | No | 2 |  |  |  |
| 18 | Neoplastic diseases (leukemia) | Yes | 1 |  |  |  |
|  |  | No | 2 |  |  |  |
| 19 | Are you taking HAART drug? | Yes | 1 |  |  |  |
|  |  | No | 2 |  |  |  |
| 20 | If Question number 19 yes then, how long | 3month of usage | 1 |  |  |  |
|  |  | 6month of usage | 2 |  |  |  |
|  |  | 9month of usage | 3 |  |  |  |
|  |  | a year of usage | 4 |  |  |  |
|  |  | Other specify | 5 |  |  |  |
| 21 | If Question number Q 19 yes drug you are taking |  | |  |  |  |
| 22 | Viral load |  | |  | 6month------------ | 9 month---------- |
| 23 | CD4 (cell/mm^3^ (%) |  | |  |  |  |
|  | | | |  |  |  |
| Nutrional data’s of participant | | | |  |  |  |
| 24 | Did you drink tea or coffee after meal? | Yes | 1 |  |  |  |
|  |  | No | 2 |  |  |  |
| 25 | If yes for tea how often tea  If yes for coffee how often |  |  |  |  |  |
|  |  |  |  |  |  |  |
| 26 | Do you eat meat | Yes | 1 |  |  |  |
|  |  | No | 2 |  |  |  |
| 27 | If answer for question 25 is yes how many times | Daily | 1 |  |  |  |
|  |  | Every two day | 2 |  |  |  |
|  |  | Every two week | 3 |  |  |  |
|  |  | Once a month | 4 |  |  |  |
| 28 | Do you eat vegetable | Yes | 1 |  |  |  |
|  |  | No | 2 |  |  |  |
| 29 | If answer for question 27 is yes how many times | Daily | 1 |  |  |  |
|  |  | Every two day | 2 |  |  |  |
|  |  | Every two week | 3 |  |  |  |
|  |  | Once a month | 4 |  |  |  |
| 30 | Have you taken iron /folate | yes | 1 |  |  |  |
|  |  | No | 2 |  |  |  |

|  |
| --- |

|  |
| --- |

Laboratory results ID.No**:______________**

| Hematologic profiles | Mean | SD | Maximum | Minimum |
| --- | --- | --- | --- | --- |
| Total leukocyte |  |  |  |  |
| Neutrophil |  |  |  |  |
| Eosinophil |  |  |  |  |
| Basophil |  |  |  |  |
| Monocyte |  |  |  |  |
| Lymphocyte |  |  |  |  |
| Erythrocyte |  |  |  |  |
| HCT (L/L) |  |  |  |  |
| MCV,fl |  |  |  |  |
| MCH,pg |  |  |  |  |
| MCHC,g/dl |  |  |  |  |
| RDW ,f l |  |  |  |  |
| Platelet |  |  |  |  |
| Viral load |  |  |  |  |
| CD4 (Cell/mm^3 (^%)) |  |  |  |  |

##

## Annex IV; Materials and reagents

- Cotton
- Pencil
- Absolute Ethanol
- UnicelDxH800 hematology analyzer
- Glove
- Test tube
- 3 or 4 ml Purple vacationer tube
- Needle
- Vacationer holder
- Alcohol swab (70%)
- Cotton balls
- Normal saline
- UnicelDxH800 reagent.
- Mixer
- New methylene /Brilliant Cresol Blue
- Antihumangloblin reagent.
- Wright satin
- Heparinized/EDTA capillary tube
- Disposable glove
- Tourniquet
- Microscope with 100X objective
- Microscope slides
- Glass test tubes
- Centrifuge
- Plastic pasture pipette
- Distilled water
- Water bath
- Oil immersion

## **Annex V; - Principles and Laboratory procedure**

**1. Sample procedure**

**Venous blood collection**

1. Assemble all necessary equipment
2. Identify the patient and label the test tubes by patient’s identification number.
3. Visually inspect and choose the arm from cephalic, basilic, and median cubital veins that was not repeatedly used for venipuncture, free of bruises, abrasions, and sites of infection.
4. Apply the tourniquet
5. Using a cotton ball saturated with 70% alcohol clean the skin in the area of the venipuncture.
6. Allow the site to dry
7. Use one hand to hold the evacuated tube or syringe and one or more fingers of the other hand to secure the skin area of the forearm below the intended venipuncture site.
8. Hold the needle with attached syringe or evacuated tube at angle of 20°about 1 to 2 inches below and in a straight line with the intended venipuncture site.
9. Gently insert the needle through the skin and into the vein.
10. Release tourniquet as soon as the blood begins to flow into the evacuated tube
11. After the desired amount of blood has been drawn place a gauze pad over the venipuncture site.
12. Withdraw the blood collecting unit with one hand and immediately press down on the gauze pad and elevate the entire arm.
13. Place a non-allergenic adhesive spot or strip over the venipuncture site
14. Mix tubes with anticoagulant by inverting the tubes several times.
15. Clean up supplies from the work area, remove gloves, and wash hands.

**2. Complete Blood Count by hematology analyzer (Unicel DHX800)**

**Principle**

The Unicel DxH 800 analyzer is a quantitative, automated hematology analyzer for in-vitro diagnostic use in screening patient populations in clinical laboratories. The blood sample, which is suspended in diluted sample, pass through the apparatus causing DC resistance. As this occur change in blood cell size is detected as the electrical pulse and blood cell count is calculated by counting pulse. The Unicel DxH 800 Analyzer provides the following: CBC, Leukocyte 5-Part Differential (Diff), Reticulocyte, and Nucleated RBC on whole blood. A cyanide-free CBC lytic reagent that lyses RBC for the white blood cell count, and works in conjunction with Coulter DxH diluent to generate a stable hemoglobin measurement and used to lyse the RBC and discriminates nucleated RBC from white blood cells.

The reticulocyte stain reagent is a cyanide-free reagent that uses a dye to stain reticulocytes. The reticulocyte-clearing reagent is a cyanide-free reagent that stabilizes the dye-reticulum complex to enhance discrimination of reticulocytes from mature RBC utilizing the volume, conductivity and scatter technology. DxH Cleaner is a cyanide-free, aldehyde-free cleaning agent that degrades residual materials so that they may be flushed from the system with the diluent.

**Specimen requirements**

About 3-4 ml of venous blood collected into EDTA tubes.

**Procedure**

- - - 1. Turn on the power switch on the front side of the analyzer.
      2. Perform quality control analysis on 3 levels of control blood material (low, normal and high) to verify that the instrument is performing within the specified ranges
      3. Specimen will be collected into EDTA (purple) vacutainer (2 or 3 ml volume).
      4. Well mix blood with EDTA and perform CBC and reticulocyte count.
      5. After the specimen processing module cycles the samples, review the sample results at the system manager.
      6. Finally print the output and register

**3. Wright stain**

Principle for Wright stain:-

Wright’s stain is a polychromatic stain consisting of a mixture of eosin and methylene Blue. When applied to blood cells, the dyes produce multiple colors based on the ionic charge of the stain and the various components of the cell. The eosin ions are negatively charged and stain basic cell components an orange to pink color. The methylene blue ions are positively charged and stain the acid cell components in varying shades of blue. The neutral components of the cell are stained by both components of the dye producing variable colors.

Procedure

1. Place the air-dried blood smears with the smeared side upward on a horizontal staining rack.

2. Cover the blood smears completely with Wright's stain solution and let it remain for 1 -3 minute.

3. Wash the stain off with running tap water until the smear appears pink.

4. Remove the stain on the back of the smears by cleaning with alcohol-moistened gauze and dry.

5. Examine under a microscope.

**Blood film finding interpretation**: - The presence of <1% fragments in the PBF is considered normal and 1-5(+1), 6–14(+2) and >15 %( +3) finding of fragmented cell in blood film is reported in graded manner in bracket. However, the finding of even a single spherocyte and nucleated RBC is considered as indicators since, they are usually absent in the blood films of healthy individuals. Reporting and interpretation for spherocyte can be as follows, 1-5, 6–19, >20% finding of in blood film reported in grade as +1, +2 and +3 respectively. Then Perform DAT.

**4. Principle of DAT;-**

DAT is performed based on the following principle is a hem agglutination test. Anti-Human Globulin Anti-IgG, -C3d; poly specific acts as a link between the antibody and/or complement coating of neighboring RBC and induces agglutination. Uncoated RBC will not agglutinate. In order for agglutination to occur, with FC portion of IgGAb or c3b or c3d component of compliment, anti-human antibody must be added to the system. This will form a bridge between antibody or compliment, coating the red cell, and antibody on the other cell causing agglutination to be visualized. In the direct form of antihuman globulin test, which is applicable in this study, sensitization occurs in vivo. Intracellular hemolysis, can be detected because of the auto antibodies alteration in membrane will be occurred; causing loss of membrane which results in spherical shape erythrocytes (spherocytosis).Even though intracellular hemolysis is there in IHA, most of the time hemolysis is found Extracellular.

**Procedure**

1. Drop of RBC will be done on test tube
2. Test cells will be washed three times with a minimum of 10 ml of saline per wash,
3. The supernatant will be removed after each wash and prepare 3% of suspension.
4. Two volumes of the anti-globulin reagent will be added to two volumes of suspension.
5. The test tube would be immediately centrifuged after thorough mixing.
6. Finally reading for presence of agglutination will be done macroscopically

**Quality Control for coombs** test

Negative Control (Red cells phenotype Rh negative)

Positive Control (Red cells phenotype Rh positive coated with anti-D)

**Procedure for preparation of positive control**

1. Collect fresh O Rho (D) positive red blood cells preferably with citrate as an anticoagulant.
2. Wash 1ml of freshly collected O Rh (D) positive red blood cells with isotonic saline at least three times.
3. After the third wash thoroughly decant the supernatant. To the cell button add 5 ml of anti-D (IgG) reagent and gently suspend the red blood cells.
4. Incubate the mixture at 37 ^o^C for 15 minutes.
5. After incubation wash the sensitized red blood cells thoroughly at least 4 to 5 times with isotonic saline.
6. Decant the supernatant thoroughly after the last wash.
7. A stabilized suspension of 5% Coombs control cells is thus

- Temperature of water bath or heating block was checked and documented
- Centrifuge equipment shall be maintained as per manufacturer’s recommendations including speed of rotation and timing device.
- Serological pipettes should be maintained as per manufacturer’s recommendations including adequate volume delivery, reduction of carryover and absence of contamination.
- The IgG coated red cells should demonstrate a minimum 2+ grade agglutination in tests with reagents containing anti-IgG.

**IHA result interpretation**

The anemia has to be Normocytic/macrocytic normochromic anemia (MCV ≥ 80 FL MCH= 27-32 evidence for the presence of hemolysis in blood film such as schistocytes including Helmet cell, Karatocyte ,bur cell, shistocytosis ,spherocytosis or nucleated RBC ([63](#_ENREF_63), [64](#_ENREF_64))DAT positive and a Reticulocyte count greater than 2.5 %. The presence of erythrophagocytosis by monocyte may be seen in severe cases and agglutinated RBC may be seen uncommonly in warm AIHA([65-67](#_ENREF_65)) as the result of such findings in blood film, with other parameters, used as evidence for the presence of IHA (74).
