# Supplementary material for: The magnitude and associated factors of immune hemolytic anemia among human immuno deficiency virus infected adults attending University of Gondar comprehensive specialized hospital north west Ethiopia 2021 GC, cross sectional study design
Source: PLoS One. 2022 Oct 6;17(10):e0274464. doi: 10.1371/journal.pone.0274464 (PMC9536541; doi:10.1371/journal.pone.0274464)
Supplement: Attachment — Submitted filename: Response to Reviewer.docx [file pone.0274464.s002.docx]

**Response to Reviewer**

**Dear reviewer 1 thank you for your constructive comments in manuscript we prepared. Accordingly I have corrected each and every of your comments.**

1. The first issue is the lack of tables on the result section. The authors have cited tables but simply did not provide them. At first, I thought that the editorial manager failed to provide me the tables but I have confirmed with the staff from PLOS ONE about the issue and the staff said that the authors did not provide the tables during submission. This sadly can happen accidentally. Unfortunately, due to this, I’m unable to verify/evaluate the data. Thus, I have to recommend the authors to resubmit the manuscript with the tables. Meanwhile, the authors perhaps could conduct some revisions on other areas of the manuscript.

**Author’s response:** Thank you dear reviewer I have added the tables as your comment

2. Were there any missing data in the study and if any, how did the authors address them?

**Author’s response:** The data were cleaned, verified and screened for any missing data

3. Flowchart of patient selection should be made that details initial total patients obtained and how many were excluded

**Author’s response**: The information for excluded study population was added

4. The 95% confidence intervals for odds ratios of several variables seem to be very wide which may indicate some variables were still not accounted for (but we still need to see the complete data/tables).

**Author’s response**: I have provided the tables as your comment for variables and the reason for high OR is due to the reason that the rare events

**Dear reviewer 2 thank you for your constructive comments in manuscript we prepared. Accordingly I have corrected each and every of your comments.**

1. I found the abstract clear and sufficient in describing the manuscript in general i.e., to describe the magnitude of IHA in PLWHA and its' associated factors.
2. The introduction might need to be shortened, there are also some repetitive statements (an example is the part where the author is explaining the factors of IHA in PLWHA) the author might need to consider rephrasing the statements so that the introduction has more fluidity.

**Author’s response**: Thank you dear reviewer I have reduced some contents of introduction as your comment

3. The methods are quite clear, the author described every variable in the manuscript. However there are some repetitive sections in the method subsection, for example in the diagnostic criteria of IHA. The authors might consider combining these sections. I also found what seems to be an inconsistent abbreviation: between UOGCSH and UOCSH.
   **Author’s response** : I have revised the methods part and I have corrected the abbreviation as UOGCSH
4. In the results section: I am not able to view the tables in the presented manuscript.
5. **Author’s response** : I have added the table as your comment
