# Supplementary material for: The magnitude and associated factors of immune hemolytic anemia among human immuno deficiency virus infected adults attending University of Gondar comprehensive specialized hospital north west Ethiopia 2021 GC, cross sectional study design
Source: PLoS One. 2022 Oct 6;17(10):e0274464. doi: 10.1371/journal.pone.0274464 (PMC9536541; doi:10.1371/journal.pone.0274464)
Supplement: Attachment — Submitted filename: Response to Reviewer revised.docx [file pone.0274464.s003.docx]

**Response to Reviewers**

**Dear reviewer 1 thank you for your constructive comments in manuscript we prepared. Accordingly we have corrected each and every of your comments.**

Reviewer #1:

1. The Authors have addressed all comments. The data presented is scientifically sound. I congratulate the Authors for a well-written manuscript. I highly recommend the manuscript for publication after the Authors address a few minor issues described below

Author’s response: Thank you for your positive comments

2. A small issue that I found is a slight data mismatch on Table 1. Here, the Authors wrote that the total sample size is 358 subjects. However, on Table 1, row for Residency (urban versus rural), the total subjects is 360. Kindly recheck and confirm the accuracy of the data.

Author’s response: Thank you for your raising this issue, this error was happened when we were preparing manuscript inserting number into tables, the total number of participant was 358( 73 rural and 285 urban).

3. On Table 2, the Authors provided total subjects on each row but this is not consistent. Some rows are not given total subjects number. I recommend in using a consistent format for Table 2. Similar issue happens on Table 4. Finally, there are some typos on Table 3 and Table 4. For examples, on table 3 row red blood cell and females; on table 4 "Ant-TB" should be corrected to "Anti-TB".

Author’s response: Thank you for your raising this issue, on table 2 meat consumption row :- the error was happened due to errors in considering the total population which was due to primarily we considered only individuals who eat meat (337) but now we corrected to peoples who consume meat as well as who did not consume meat (358). On table 2, other medication row the total population to be considered was 205. We have corrected the typos error as anti-TB., I made it consistent

4. On Table 4 for the AOR, there should be a p value for meat consumption less often/weak {0.98(0.19 5.0)} as well. Kindly recheck the table for accuracy.

Author’s response: Thank you for your raising this issue, I missed the coma in between (0.19, 5)

5. All other data and statistical analysis appear to be correct from my view. English language is of good quality and adequate literatures have been cited in the manuscript

Author’s response: Thank you for your appreciation and good comments

**Dear reviewer 2 thank you for your constructive comments in manuscript we prepared. Accordingly we have corrected each and every of your comments.**

Reviewer #2:

1. Thank you for sending the revisions and thank you for including the tables. However we believe that further revision is still needed for the manuscript.

Author’s response: Thank you for your positive and constructive comments, I have done your comments intensively.

2. There are still typographical and grammatical errors here and there (for example "Jehovah Wittiness" and "Navirapine")

Author’s response: Thank you for your raising this issue, English language professionals corrected all grammatical and typographical comments as you raised (“Jehovah's Witness” and nevirapine

3. The author might still also need to review the abbreviations used because some inconsistencies are found within the manuscript.

Author’s response: Thank you for your raising this issue, we have rechecked all abbreviation and removed inconsistency by revising whole document

4. The authors included the formula for sample calculations, we don't think this is necessary for the published version of the manuscript.

Author’s response: Thank you for your raising this issue, we have removed the sample size calculation formulas from manuscript

5. The newly added table formatting should also be reviewed for its formatting, borders and presentation (Table 2, 3, 4).

Author’s response: Thank you for your raising this issue, thank you we had made changes on border line

6. For the discussion, I think that the authors have done well to present the magnitude of IHA in PLWHA, however I think that could add more comparisons to other studies regarding the findings. The authors mainly compared the study with those that are done in Ethiopia and Nigeria

Author’s response: Thank you for your raising this issue, because we didn’t find the study related to IHA burden on other nations in quantitative manner
