# Supplementary material for: The magnitude and associated factors of immune hemolytic anemia among human immuno deficiency virus infected adults attending University of Gondar comprehensive specialized hospital north west Ethiopia 2021 GC, cross sectional study design
Source: PLoS One. 2022 Oct 6;17(10):e0274464. doi: 10.1371/journal.pone.0274464 (PMC9536541; doi:10.1371/journal.pone.0274464)
Supplement: Peer review history

# Peer review: Decision Letter 0

**DOI:** 10.1371/journal.pone.0274464.r001

**Authors:** Evy Yunihastuti

25 Jan 2022

PONE-D-21-33300Magnitude and associated factors of  immune hemolytic anemia  among Human Immunodeficiency virus infected adults at University of Gondar specialized hospital North west Ethiopia, cross sectional studyPLOS ONE

Dear Dr. Kabede,

Thank you for submitting your manuscript to PLOS ONE. After careful consideration, we feel that it has merit but does not fully meet PLOS ONE’s publication criteria as it currently stands. Therefore, we invite you to submit a revised version of the manuscript that addresses the points raised during the review process.

 Please provide appropriate flowchart and tables as needed.

Please submit your revised manuscript by Mar 11 2022 11:59PM. If you will need more time than this to complete your revisions, please reply to this message or contact the journal office at plosone@plos.org. When you're ready to submit your revision, log on to https://www.editorialmanager.com/pone/ and select the 'Submissions Needing Revision' folder to locate your manuscript file.

Please include the following items when submitting your revised manuscript:

- A rebuttal letter that responds to each point raised by the academic editor and reviewer(s). You should upload this letter as a separate file labeled 'Response to Reviewers'.
- A marked-up copy of your manuscript that highlights changes made to the original version. You should upload this as a separate file labeled 'Revised Manuscript with Track Changes'.
- An unmarked version of your revised paper without tracked changes. You should upload this as a separate file labeled 'Manuscript'.

If you would like to make changes to your financial disclosure, please include your updated statement in your cover letter. Guidelines for resubmitting your figure files are available below the reviewer comments at the end of this letter.

If applicable, we recommend that you deposit your laboratory protocols in protocols.io to enhance the reproducibility of your results. Protocols.io assigns your protocol its own identifier (DOI) so that it can be cited independently in the future. For instructions see: https://journals.plos.org/plosone/s/submission-guidelines#loc-laboratory-protocols. Additionally, PLOS ONE offers an option for publishing peer-reviewed Lab Protocol articles, which describe protocols hosted on protocols.io. Read more information on sharing protocols at https://plos.org/protocols?utm_medium=editorial-email&utm_source=authorletters&utm_campaign=protocols.

We look forward to receiving your revised manuscript.

Kind regards,

Evy Yunihastuti, MD

Academic Editor

PLOS ONE

Journal Requirements:

When submitting your revision, we need you to address these additional requirements.

1. Please ensure that your manuscript meets PLOS ONE's style requirements, including those for file naming. The PLOS ONE style templates can be found at 

https://journals.plos.org/plosone/s/file?id=wjVg/PLOSOne_formatting_sample_main_body.pdf and 

https://journals.plos.org/plosone/s/file?id=ba62/PLOSOne_formatting_sample_title_authors_affiliations.pdf

2. PLOS requires an ORCID iD for the corresponding author in Editorial Manager on papers submitted after December 6th, 2016. Please ensure that you have an ORCID iD and that it is validated in Editorial Manager. To do this, go to ‘Update my Information’ (in the upper left-hand corner of the main menu), and click on the Fetch/Validate link next to the ORCID field. This will take you to the ORCID site and allow you to create a new iD or authenticate a pre-existing iD in Editorial Manager. Please see the following video for instructions on linking an ORCID iD to your Editorial Manager account: https://www.youtube.com/watch?v=_xcclfuvtxQ

3. Please include your full ethics statement in the ‘Methods’ section of your manuscript file. In your statement, please include the full name of the IRB or ethics committee who approved or waived your study, as well as whether or not you obtained informed written or verbal consent. If consent was waived for your study, please include this information in your statement as well. 

[Note: HTML markup is below. Please do not edit.]

Reviewers' comments:

Reviewer's Responses to Questions

**Comments to the Author**

1. Is the manuscript technically sound, and do the data support the conclusions?

The manuscript must describe a technically sound piece of scientific research with data that supports the conclusions. Experiments must have been conducted rigorously, with appropriate controls, replication, and sample sizes. The conclusions must be drawn appropriately based on the data presented. 

Reviewer #1: Partly

Reviewer #2: Yes

**********

2. Has the statistical analysis been performed appropriately and rigorously? 

Reviewer #1: I Don't Know

Reviewer #2: I Don't Know

**********

3. Have the authors made all data underlying the findings in their manuscript fully available?

The PLOS Data policy requires authors to make all data underlying the findings described in their manuscript fully available without restriction, with rare exception (please refer to the Data Availability Statement in the manuscript PDF file). The data should be provided as part of the manuscript or its supporting information, or deposited to a public repository. For example, in addition to summary statistics, the data points behind means, medians and variance measures should be available. If there are restrictions on publicly sharing data—e.g. participant privacy or use of data from a third party—those must be specified.

Reviewer #1: No

Reviewer #2: No

**********

4. Is the manuscript presented in an intelligible fashion and written in standard English?

PLOS ONE does not copyedit accepted manuscripts, so the language in submitted articles must be clear, correct, and unambiguous. Any typographical or grammatical errors should be corrected at revision, so please note any specific errors here.

Reviewer #1: Yes

Reviewer #2: Yes

**********

5. Review Comments to the Author

Please use the space provided to explain your answers to the questions above. You may also include additional comments for the author, including concerns about dual publication, research ethics, or publication ethics. (Please upload your review as an attachment if it exceeds 20,000 characters)

Reviewer #1: Dear Authors and Editors, I have read the manuscript submitted by the authors with great interest. From the manuscript, the authors conducted a cross-sectional study to determine factors associated with immune hemolytic anemia in HIV adult patients. The study is methodologically sound and the sample size appears to be adequate. Furthermore, the manuscript has adequate language quality.

I do believe the manuscript is appropriate for publication in PLOS ONE, however, there are several issues/information that should be addressed/clarified first:

1. The first issue is the lack of tables on the result section. The authors have cited tables but simply did not provide them. At first, I thought that the editorial manager failed to provide me the tables but I have confirmed with the staff from PLOS ONE about the issue and the staff said that the authors did not provide the tables during submission. This sadly can happen accidentally. Unfortunately, due to this, I’m unable to verify/evaluate the data. Thus, I have to recommend the authors to resubmit the manuscript with the tables. Meanwhile, the authors perhaps could conduct some revisions on other areas of the manuscript.

2. Were there any missing data in the study and if any, how did the authors address them?

3. Flowchart of patient selection should be made that details initial total patients obtained and how many were excluded.

4. The 95% confidence intervals for odds ratios of several variables seem to be very wide which may indicate some variables were still not accounted for (but we still need to see the complete data/tables).

Reviewer #2: Thank you for submitting the manuscript, I believe the paper is important and relevant, especially in terms of increasing awareness regarding the matter (IHA in PLWHA).

1. I found the abstract clear and sufficient in describing the manuscript in general i.e., to describe the magnitude of IHA in PLWHA and its' associated factors.

2. The introduction might need to be shortened, there are also some repetitive statements (an example is the part where the author is explaining the factors of IHA in PLWHA) The author might need to consider rephrasing the statements so that the introduction has more fluidity.

3. The methods are quite clear, the author described every variable in the manuscript. However there are some repetitive sections in the method subsection, for example in the diagnostic criteria of IHA. The authors might consider combining these sections. I also found what seems to be an inconsistent abbreviation: between UOGCSH and UOCSH.

4. In the results section: I am not able to view the tables in the presented manuscript.

5. The author concluded the manuscript well, especially in terms of recommending routine evaluation of IHA in PLWHA.

6. The written English needs to be proofread by a native English speaking proofreading service in order to make the manuscript more clear.

**********

6. PLOS authors have the option to publish the peer review history of their article (what does this mean?). If published, this will include your full peer review and any attached files.

If you choose “no”, your identity will remain anonymous but your review may still be made public.

**Do you want your identity to be public for this peer review?** For information about this choice, including consent withdrawal, please see our Privacy Policy.

Reviewer #1: No

Reviewer #2: No

[NOTE: If reviewer comments were submitted as an attachment file, they will be attached to this email and accessible via the submission site. Please log into your account, locate the manuscript record, and check for the action link "View Attachments". If this link does not appear, there are no attachment files.]

While revising your submission, please upload your figure files to the Preflight Analysis and Conversion Engine (PACE) digital diagnostic tool, https://pacev2.apexcovantage.com/. PACE helps ensure that figures meet PLOS requirements. To use PACE, you must first register as a user. Registration is free. Then, login and navigate to the UPLOAD tab, where you will find detailed instructions on how to use the tool. If you encounter any issues or have any questions when using PACE, please email PLOS at figures@plos.org. Please note that Supporting Information files do not need this step.

# Author comment: Author response to Decision Letter 0

**DOI:** 10.1371/journal.pone.0274464.r002

23 Mar 2022

Response to Reviewer

Dear reviewer 1 thank you for your constructive comments in manuscript we prepared. Accordingly I have corrected each and every of your comments.

1. The first issue is the lack of tables on the result section. The authors have cited tables but simply did not provide them. At first, I thought that the editorial manager failed to provide me the tables but I have confirmed with the staff from PLOS ONE about the issue and the staff said that the authors did not provide the tables during submission. This sadly can happen accidentally. Unfortunately, due to this, I’m unable to verify/evaluate the data. Thus, I have to recommend the authors to resubmit the manuscript with the tables. Meanwhile, the authors perhaps could conduct some revisions on other areas of the manuscript.

Author’s response: Thank you dear reviewer I have added the tables as your comment

2. Were there any missing data in the study and if any, how did the authors address them?

Author’s response: The data were cleaned, verified and screened for any missing data

3. Flowchart of patient selection should be made that details initial total patients obtained and how many were excluded

Author’s response: The information for excluded study population was added

4. The 95% confidence intervals for odds ratios of several variables seem to be very wide which may indicate some variables were still not accounted for (but we still need to see the complete data/tables).

Author’s response: I have provided the tables as your comment for variables and the reason for high OR is due to the reason that the rare events

Dear reviewer 2 thank you for your constructive comments in manuscript we prepared. Accordingly I have corrected each and every of your comments.

1. I found the abstract clear and sufficient in describing the manuscript in general i.e., to describe the magnitude of IHA in PLWHA and its' associated factors.

2. The introduction might need to be shortened, there are also some repetitive statements (an example is the part where the author is explaining the factors of IHA in PLWHA) the author might need to consider rephrasing the statements so that the introduction has more fluidity.

Author’s response: Thank you dear reviewer I have reduced some contents of introduction as your comment

3. The methods are quite clear, the author described every variable in the manuscript. However there are some repetitive sections in the method subsection, for example in the diagnostic criteria of IHA. The authors might consider combining these sections. I also found what seems to be an inconsistent abbreviation: between UOGCSH and UOCSH.

Author’s response : I have revised the methods part and I have corrected the abbreviation as UOGCSH 

4. In the results section: I am not able to view the tables in the presented manuscript.

5. Author’s response : I have added the table as your comment

Attachment Submitted filename: Response to Reviewer.docx Click here for additional data file.

# Peer review: Decision Letter 1

**DOI:** 10.1371/journal.pone.0274464.r003

**Authors:** Evy Yunihastuti

16 May 2022

PONE-D-21-33300R1Magnitude and associated factors of  immune hemolytic anemia  among Human Immunodeficiency virus infected adults at University of Gondar specialized hospital Northwest EthiopiaPLOS ONE

Dear Dr. Kebede,

Thank you for submitting your manuscript to PLOS ONE. After careful consideration, we feel that it has merit but does not fully meet PLOS ONE’s publication criteria as it currently stands. Therefore, we invite you to submit a revised version of the manuscript that addresses the points raised during the review process.

Please submit your revised manuscript by Jun 30 2022 11:59PM. If you will need more time than this to complete your revisions, please reply to this message or contact the journal office at plosone@plos.org. When you're ready to submit your revision, log on to https://www.editorialmanager.com/pone/ and select the 'Submissions Needing Revision' folder to locate your manuscript file.

Please include the following items when submitting your revised manuscript:

- A rebuttal letter that responds to each point raised by the academic editor and reviewer(s). You should upload this letter as a separate file labeled 'Response to Reviewers'.
- A marked-up copy of your manuscript that highlights changes made to the original version. You should upload this as a separate file labeled 'Revised Manuscript with Track Changes'.
- An unmarked version of your revised paper without tracked changes. You should upload this as a separate file labeled 'Manuscript'.

If you would like to make changes to your financial disclosure, please include your updated statement in your cover letter. Guidelines for resubmitting your figure files are available below the reviewer comments at the end of this letter.

If applicable, we recommend that you deposit your laboratory protocols in protocols.io to enhance the reproducibility of your results. Protocols.io assigns your protocol its own identifier (DOI) so that it can be cited independently in the future. For instructions see: https://journals.plos.org/plosone/s/submission-guidelines#loc-laboratory-protocols. Additionally, PLOS ONE offers an option for publishing peer-reviewed Lab Protocol articles, which describe protocols hosted on protocols.io. Read more information on sharing protocols at https://plos.org/protocols?utm_medium=editorial-email&utm_source=authorletters&utm_campaign=protocols.

We look forward to receiving your revised manuscript.

Kind regards,

Evy Yunihastuti, MD

Academic Editor

PLOS ONE

Journal Requirements:

Please review your reference list to ensure that it is complete and correct. If you have cited papers that have been retracted, please include the rationale for doing so in the manuscript text, or remove these references and replace them with relevant current references. Any changes to the reference list should be mentioned in the rebuttal letter that accompanies your revised manuscript. If you need to cite a retracted article, indicate the article’s retracted status in the References list and also include a citation and full reference for the retraction notice.

[Note: HTML markup is below. Please do not edit.]

Reviewers' comments:

Reviewer's Responses to Questions

**Comments to the Author**

1. If the authors have adequately addressed your comments raised in a previous round of review and you feel that this manuscript is now acceptable for publication, you may indicate that here to bypass the “Comments to the Author” section, enter your conflict of interest statement in the “Confidential to Editor” section, and submit your "Accept" recommendation.

Reviewer #1: All comments have been addressed

Reviewer #2: (No Response)

**********

2. Is the manuscript technically sound, and do the data support the conclusions?

The manuscript must describe a technically sound piece of scientific research with data that supports the conclusions. Experiments must have been conducted rigorously, with appropriate controls, replication, and sample sizes. The conclusions must be drawn appropriately based on the data presented. 

Reviewer #1: Partly

Reviewer #2: Partly

**********

3. Has the statistical analysis been performed appropriately and rigorously? 

Reviewer #1: Yes

Reviewer #2: No

**********

4. Have the authors made all data underlying the findings in their manuscript fully available?

The PLOS Data policy requires authors to make all data underlying the findings described in their manuscript fully available without restriction, with rare exception (please refer to the Data Availability Statement in the manuscript PDF file). The data should be provided as part of the manuscript or its supporting information, or deposited to a public repository. For example, in addition to summary statistics, the data points behind means, medians and variance measures should be available. If there are restrictions on publicly sharing data—e.g. participant privacy or use of data from a third party—those must be specified.

Reviewer #1: Yes

Reviewer #2: Yes

**********

5. Is the manuscript presented in an intelligible fashion and written in standard English?

PLOS ONE does not copyedit accepted manuscripts, so the language in submitted articles must be clear, correct, and unambiguous. Any typographical or grammatical errors should be corrected at revision, so please note any specific errors here.

Reviewer #1: Yes

Reviewer #2: Yes

**********

6. Review Comments to the Author

Please use the space provided to explain your answers to the questions above. You may also include additional comments for the author, including concerns about dual publication, research ethics, or publication ethics. (Please upload your review as an attachment if it exceeds 20,000 characters)

Reviewer #1: 1. The Authors have addressed all comments. The data presented is scientifically sound. I congratulate the Authors for a well-written manuscript. I highly recommend the manuscript for publication after the Authors address a few minor issues described below

2. A small issue that I found is a slight data mismatch on Table 1. Here, the Authors wrote that the total sample size is 358 subjects. However, on Table 1, row for Residency (urban versus rural), the total subjects is 360. Kindly recheck and confirm the accuracy of the data.

3. On Table 2., the Authors provided total subjects on each row but this is not consistent. Some rows are not given total subjects number. I recommend in using a consistent format for Table 2. Similar issue happens on Table 4. Finally, there are some typos on Table 3 and Table 4. For examples, on table 3 row red blood cell and females; on table 4 "Ant-TB" should be corrected to "Anti-TB"

4. On Table 4 for the AOR, there should be a p value for meat consumption less often/weak {0.98(0.19 5.0)} as well. Kindly recheck the table for accuracy.

5. All other data and statistical analysis appear to be correct from my view. English language is of good quality and adequate literatures have been cited in the manuscript

Reviewer #2: Thank you for sending the revisions and thank you for including the tables. However I believe that further revision is still needed for the manuscript.

There are still typographical and grammatical errors here and there (for example "Jehovah Wittiness" and "Navirapine")

The author might still also need to review the abbreviations used because some inconsistencies are found within the manuscript.

The authors included the formula for sample calculations, i don't think this is necessary for the published version of the manuscript.

The newly added table formatting should also be reviewed for its formatting, borders and presentation (Table 2, 3, 4).

For the discussion, I think that the authors have done well to present the magnitude of IHA in PLWHA, however I think that could add more comparisons to other studies regarding the findings. The authors mainly compared the study with those that are done in Ethiopia and Nigeria.

**********

7. PLOS authors have the option to publish the peer review history of their article (what does this mean?). If published, this will include your full peer review and any attached files.

If you choose “no”, your identity will remain anonymous but your review may still be made public.

**Do you want your identity to be public for this peer review?** For information about this choice, including consent withdrawal, please see our Privacy Policy.

Reviewer #1: **Yes: **Ikhwan Rinaldi

Reviewer #2: **Yes: **Andhika Rachman, MD, PhD.

[NOTE: If reviewer comments were submitted as an attachment file, they will be attached to this email and accessible via the submission site. Please log into your account, locate the manuscript record, and check for the action link "View Attachments". If this link does not appear, there are no attachment files.]

While revising your submission, please upload your figure files to the Preflight Analysis and Conversion Engine (PACE) digital diagnostic tool, https://pacev2.apexcovantage.com/. PACE helps ensure that figures meet PLOS requirements. To use PACE, you must first register as a user. Registration is free. Then, login and navigate to the UPLOAD tab, where you will find detailed instructions on how to use the tool. If you encounter any issues or have any questions when using PACE, please email PLOS at figures@plos.org. Please note that Supporting Information files do not need this step.

# Author comment: Author response to Decision Letter 1

**DOI:** 10.1371/journal.pone.0274464.r004

20 May 2022

Response to Reviewers

Dear reviewer 1 thank you for your constructive comments in manuscript we prepared. Accordingly we have corrected each and every of your comments.

Reviewer #1: 

1. The Authors have addressed all comments. The data presented is scientifically sound. I congratulate the Authors for a well-written manuscript. I highly recommend the manuscript for publication after the Authors address a few minor issues described below

Author’s response: Thank you for your positive comments

2. A small issue that I found is a slight data mismatch on Table 1. Here, the Authors wrote that the total sample size is 358 subjects. However, on Table 1, row for Residency (urban versus rural), the total subjects is 360. Kindly recheck and confirm the accuracy of the data.

Author’s response: Thank you for your raising this issue, this error was happened when we were preparing manuscript inserting number into tables, the total number of participant was 358( 73 rural and 285 urban).

3. On Table 2, the Authors provided total subjects on each row but this is not consistent. Some rows are not given total subjects number. I recommend in using a consistent format for Table 2. Similar issue happens on Table 4. Finally, there are some typos on Table 3 and Table 4. For examples, on table 3 row red blood cell and females; on table 4 "Ant-TB" should be corrected to "Anti-TB".

Author’s response: Thank you for your raising this issue, on table 2 meat consumption row :- the error was happened due to errors in considering the total population which was due to primarily we considered only individuals who eat meat (337) but now we corrected to peoples who consume meat as well as who did not consume meat (358). On table 2, other medication row the total population to be considered was 205. We have corrected the typos error as anti-TB., I made it consistent

4. On Table 4 for the AOR, there should be a p value for meat consumption less often/weak {0.98(0.19 5.0)} as well. Kindly recheck the table for accuracy.

Author’s response: Thank you for your raising this issue, I missed the coma in between (0.19, 5)

5. All other data and statistical analysis appear to be correct from my view. English language is of good quality and adequate literatures have been cited in the manuscript

 Author’s response: Thank you for your appreciation and good comments

Dear reviewer 2 thank you for your constructive comments in manuscript we prepared. Accordingly we have corrected each and every of your comments.

Reviewer #2: 

1. Thank you for sending the revisions and thank you for including the tables. However we believe that further revision is still needed for the manuscript.

Author’s response: Thank you for your positive and constructive comments, I have done your comments intensively.

2. There are still typographical and grammatical errors here and there (for example "Jehovah Wittiness" and "Navirapine")

 Author’s response: Thank you for your raising this issue, English language professionals corrected all grammatical and typographical comments as you raised (“Jehovah's Witness” and nevirapine

3. The author might still also need to review the abbreviations used because some inconsistencies are found within the manuscript.

Author’s response: Thank you for your raising this issue, we have rechecked all abbreviation and removed inconsistency by revising whole document

4. The authors included the formula for sample calculations, we don't think this is necessary for the published version of the manuscript.

Author’s response: Thank you for your raising this issue, we have removed the sample size calculation formulas from manuscript

5. The newly added table formatting should also be reviewed for its formatting, borders and presentation (Table 2, 3, 4).

Author’s response: Thank you for your raising this issue, thank you we had made changes on border line

6. For the discussion, I think that the authors have done well to present the magnitude of IHA in PLWHA, however I think that could add more comparisons to other studies regarding the findings. The authors mainly compared the study with those that are done in Ethiopia and Nigeria

Author’s response: Thank you for your raising this issue, because we didn’t find the study related to IHA burden on other nations in quantitative manner

Attachment Submitted filename: Response to Reviewer revised.docx Click here for additional data file.

# Peer review: Decision Letter 2

**DOI:** 10.1371/journal.pone.0274464.r005

**Authors:** Evy Yunihastuti

30 Aug 2022

Magnitude and associated factors of  immune hemolytic anemia  among Human Immunodeficiency virus infected adults at University of Gondar specialized hospital Northwest Ethiopia

PONE-D-21-33300R2

Dear Dr. Kebede,

We’re pleased to inform you that your manuscript has been judged scientifically suitable for publication and will be formally accepted for publication once it meets all outstanding technical requirements.

Within one week, you’ll receive an e-mail detailing the required amendments. When these have been addressed, you’ll receive a formal acceptance letter and your manuscript will be scheduled for publication.

An invoice for payment will follow shortly after the formal acceptance. To ensure an efficient process, please log into Editorial Manager at http://www.editorialmanager.com/pone/, click the 'Update My Information' link at the top of the page, and double check that your user information is up-to-date. If you have any billing related questions, please contact our Author Billing department directly at authorbilling@plos.org.

If your institution or institutions have a press office, please notify them about your upcoming paper to help maximize its impact. If they’ll be preparing press materials, please inform our press team as soon as possible -- no later than 48 hours after receiving the formal acceptance. Your manuscript will remain under strict press embargo until 2 pm Eastern Time on the date of publication. For more information, please contact onepress@plos.org.

Kind regards,

Evy Yunihastuti, MD

Academic Editor

PLOS ONE

Additional Editor Comments (optional):

Reviewers' comments:

Reviewer's Responses to Questions

**Comments to the Author**

1. If the authors have adequately addressed your comments raised in a previous round of review and you feel that this manuscript is now acceptable for publication, you may indicate that here to bypass the “Comments to the Author” section, enter your conflict of interest statement in the “Confidential to Editor” section, and submit your "Accept" recommendation.

Reviewer #1: All comments have been addressed

Reviewer #2: All comments have been addressed

**********

2. Is the manuscript technically sound, and do the data support the conclusions?

The manuscript must describe a technically sound piece of scientific research with data that supports the conclusions. Experiments must have been conducted rigorously, with appropriate controls, replication, and sample sizes. The conclusions must be drawn appropriately based on the data presented. 

Reviewer #1: Yes

Reviewer #2: Yes

**********

3. Has the statistical analysis been performed appropriately and rigorously? 

Reviewer #1: Yes

Reviewer #2: Yes

**********

4. Have the authors made all data underlying the findings in their manuscript fully available?

The PLOS Data policy requires authors to make all data underlying the findings described in their manuscript fully available without restriction, with rare exception (please refer to the Data Availability Statement in the manuscript PDF file). The data should be provided as part of the manuscript or its supporting information, or deposited to a public repository. For example, in addition to summary statistics, the data points behind means, medians and variance measures should be available. If there are restrictions on publicly sharing data—e.g. participant privacy or use of data from a third party—those must be specified.

Reviewer #1: Yes

Reviewer #2: Yes

**********

5. Is the manuscript presented in an intelligible fashion and written in standard English?

PLOS ONE does not copyedit accepted manuscripts, so the language in submitted articles must be clear, correct, and unambiguous. Any typographical or grammatical errors should be corrected at revision, so please note any specific errors here.

Reviewer #1: Yes

Reviewer #2: Yes

**********

6. Review Comments to the Author

Please use the space provided to explain your answers to the questions above. You may also include additional comments for the author, including concerns about dual publication, research ethics, or publication ethics. (Please upload your review as an attachment if it exceeds 20,000 characters)

Reviewer #1: Dear Authors, thankyou for the second revision. I have no further comments for the manuscript and I recommend the manuscript to enter galley proof/production stage for publication.

I wish the best for all of Authors' works and future endeavors.

Reviewer #2: Thank you for the revision and for addressing the previous comments thoroughly. I believe that the article provide interesting facts and is presented appropriately.

**********

7. PLOS authors have the option to publish the peer review history of their article (what does this mean?). If published, this will include your full peer review and any attached files.

If you choose “no”, your identity will remain anonymous but your review may still be made public.

**Do you want your identity to be public for this peer review?** For information about this choice, including consent withdrawal, please see our Privacy Policy.

Reviewer #1: **Yes: **Ikhwan Rinaldi

Reviewer #2: **Yes: **Andhika Rachman

**********

# Editor report: Acceptance letter

**DOI:** 10.1371/journal.pone.0274464.r006

**Authors:** Evy Yunihastuti

18 Sep 2022

PONE-D-21-33300R2 

The Magnitude and associated factors of Immune hemolytic anemia among human immuno deficiency virus infected adults attending University of Gondar comprehensive specialized hospital north west Ethiopia 2021 GC,cross sectional study design 

Dear Dr. Kebede:

I'm pleased to inform you that your manuscript has been deemed suitable for publication in PLOS ONE. Congratulations! Your manuscript is now with our production department. 

If your institution or institutions have a press office, please let them know about your upcoming paper now to help maximize its impact. If they'll be preparing press materials, please inform our press team within the next 48 hours. Your manuscript will remain under strict press embargo until 2 pm Eastern Time on the date of publication. For more information please contact onepress@plos.org.

If we can help with anything else, please email us at plosone@plos.org. 

Thank you for submitting your work to PLOS ONE and supporting open access. 

Kind regards, 

PLOS ONE Editorial Office Staff

on behalf of

Dr. Evy Yunihastuti 

Academic Editor

PLOS ONE
